# Distribution of *KRAS*, *DDR2*, and *TP53* gene mutations in lung cancer: An analysis of Iranian patients

**DOI:** 10.1371/journal.pone.0200633

**Published:** 2018-07-26

**Authors:** Zahra Fathi, Seyed Ali Javad Mousavi, Raheleh Roudi, Farideh Ghazi

**Affiliations:** 1 Department of Medical Genetics and Molecular Biology, Faculty of Medicine, Iran University of Medical Sciences, Tehran, Iran; 2 Minimally Invasive Surgery Research Center, Iran University of Medical Sciences, Tehran, Iran; 3 Oncopathology Research Center, Iran University of Medical Sciences, Tehran, Iran; Virginia Commonwealth University, UNITED STATES

## Abstract

**Purpose:**

Lung cancer is the deadliest known cancer in the world, with the highest number of mutations in proto-oncogenes and tumor suppressor genes. Therefore, this study was conducted to determine the status of hotspot regions in *DDR2* and *KRAS* genes for the first time, as well as in *TP53* gene, in lung cancer patients within the Iranian population.

**Experimental design:**

The mutations in exon 2 of *KRAS*, exon 18 of *DDR2*, and exons 5–6 of *TP53* genes were screened in lung cancer samples, including non-small cell lung cancer (NSCLC) and small cell lung cancer (SCLC) using PCR and sequencing techniques.

**Results:**

Analysis of the *KRAS* gene showed only a *G12C* variation in one large cell carcinoma (LCC) patient, whereas variants were not found in adenocarcinoma (ADC) and squamous cell carcinoma (SCC) cases. The *Q808H* variation in the *DDR2* gene was detected in one SCC sample, while no variant was seen in the ADC and LCC subtypes. Variations in the *TP53* gene were seen in all NSCLC subtypes, including six ADC (13.63%), seven SCC (15.9%) and two LCC (4.54%). Forty-eight variants were found in the *TP53* gene. Of these, 15 variants were found in coding regions *V147A*, *V157F*, *Q167Q*, *D186G*, *H193R*, *T211T*, *F212L* and *P222P*, 33 variants in intronic regions rs1625895 (HGVS: *c*.*672+62A>G*), rs766856111 (HGVS: *c*.*672+6G>A*) and two new variants (*c*.*560-12A>G* and *c*.*672+86T>C*).

**Conclusions:**

In conclusion, *KRAS*, *DDR2*, and *TP53* variants were detected in 2%, 2.17% and 79.54% of all cases, respectively. The frequency of *DDR2* mutation is nearly close to other studies, while *KRAS* and *TP53* mutation frequencies are lower and higher than other populations, respectively. Three new putative pathogenic variants, for the first time, have been detected in Iranian patients with lung cancer, including *Q808H* in *DDR2*, *F212L*, and *D186G* in coding regions of *TP53*. In addition, we observed five novel benign variants, including *Q167Q*, *P222P* and *T211T* in coding sequence, and *c*.*560-12A>G* and *c*.*672+86T>C*, in intronic region of *TP53*. Mutations of *KRAS* and *DDR2* were found in LCC and SCC subtypes, respectively, whereas mutations of *TP53* were seen in SCC and ADC subtypes with higher frequencies and LCC subtype with lower frequency. Therefore, Iranian lung cancer patients can benefit from mutational analysis before starting the conventional treatment. A better understanding of the biology of these genes and their mutations will be critical for developing future targeted therapies.

## Introduction

Lung cancer is the leading cause of cancer-related death in both men and women worldwide. Non-small cell lung cancer (NSCLC), with an incidence of 80% to 85%, is the most common type of lung cancer [1]. Lung cancer is often diagnosed when a person is in advanced stages of the disease and the prognosis is poor [2].

Many efforts have been made to treat patients with lung cancer. Surgery, chemotherapy, radiotherapy, and targeted therapies are conventional lung cancer treatments [3]. Targeted therapies with tyrosine kinase inhibitors (TKIs) comprise epidermal growth factor receptor (EGFR) inhibitors, such as erlotinib or gefitinib, and anaplastic lymphoma kinase (ALK) inhibitors, such as crizotinib [4, 5]. Considering the high mortality and morbidity rates of lung cancer and the emergence of drug resistance to chemoradiotherapy regimens and TKIs, determining targetable genetic changes is of paramount importance [6].

Research has shown that the genetic variation in lung cancer is higher than that of other cancers [7]. The *DDR2* gene, which is located on the long arm of chromosome 1 (1q23.3) is a tyrosine kinase receptor that plays a critical role in cellular connectivity, survival, migration and cell proliferation [8]. In tumor cells, driver mutations in kinase domain activation loops, autoinhibitory juxtamembrane regions, and ligand binding domains, can interrupt kinase function and initiate pro‑migratory and pro‑invasive cascades [9]. A substitution of serine to arginine at position 768 (*S768R*) of exon 18 has been reported as the most common mutation in the *DDR2* gene [8, 10]. In one study, Hammerman et al. found that *DDR2* mutations account for nearly 4% of squamous cell carcinoma (SCC) subtype [8]. Further evaluations in Korea, China, and France populations revealed that the frequencies of *DDR2* mutations were 2%, 4.6%, and 4% in SCC, respectively [10–12]. However, Kenmotsu et al. and Yashima et al. did not find any mutations in *DDR2* gene of Japanese SCC patients [13, 14]. In addition, despite the broader range of mutated genes in SCC, there is no effective targeted treatment for this subtype [15–17]. Some studies have shown that the targeting of *DDR2* by FDA-approved kinase inhibitors including dasatinib, imatinib, nilotinib, and ponatinib can suppress the proliferation of this gene in mutated cancer cell lines [18, 19]. Dramatic response to dasatinib has been reported in SCC patients harboring *S768R* mutations in exon 18 of *DDR2*, and thus this region has been introduced as a valuable molecular target of TKIs in these patients [10, 20].

*KRAS* proto-oncogene (12p12.1) is a GTPase that is located on the downstream pathway of the tyrosine kinase receptors and involved in cell growth, differentiation, and apoptosis. Investigations of *KRAS* status in NSCLC patients revealed a wide spectrum of mutations in different countries: 8.4% in China, 21% in Japan, 27% in Greece and Italy, 29% in France, and 43.3% in Spain [21–26]. The most prevalent mutated region of *KRAS* in lung cancer is codon 12 (exon 2) with 75% frequency, whereas mutations in other regions of *KRAS* are less frequent including codon 13 (exon 2) and codon 63 (exon 3) [27, 28]. A previous meta-analysis demonstrated that the presence of *KRAS* mutations was a negative prognostic factor for the overall survival of patients with lung cancer, but a more recent study showed that only the presence of *KRAS* mutations in exon 2 had a predictive value in adenocarcinoma (ADC) patients [27, 29]. Targeted therapies with TKIs have been effective in ADC, but the presence of *KRAS* mutations induces resistance to treatment with EGFR-independent mechanisms [6]. The RAS/MAPK pathway, with the key component of *KRAS*, is one of the major signaling networks linking to EGFR signaling. Hence, mutations in downstream effectors of EGFR signaling could lead to resistance to EGFR inhibitors [30]. Moreover, the response to TKIs varies among lung cancer patients with *KRAS* mutations and may be affected by such factors as coexistence of mutations in tumor suppressor genes (*TP53* or *PTEN*) [31, 32]. Simultaneous analysis of *KRAS* and *TP53* mutations has an important role in determining the prognosis and appropriate treatment strategies for lung cancer patients [33]. The gene *TP53*, 17p13.1, encodes a tumor suppressor protein that plays a role in regulating the cell cycle. In genomic damage, *TP53* plays an anti-cancer role by preventing and suppressing abnormal cell growth by cell cycle arrest, DNA repair, control of metabolism, and apoptosis. Mutations within the *TP53* gene itself or mutations of downstream mediators of *TP53* lead to inactivation of its function [34, 35]. Prevalence of *TP53* gene mutation accounts for nearly 39% of ADC, 51% of SCC, 68% of large cell carcinoma (LCC), and 80% of small cell lung cancer (SCLC) [34, 36]. In addition, a frequent variation has been found in *TP53* mutations in lung cancer patients with different ethnicities [37].

Previous studies have shown that *TP53* gene in exons 5 to 8 has a considerably higher mutation rate and exons 5–6 have been identified as the mutational hotspot regions [38, 39]. A more recent and comprehensive study by Baugh et al determined a list of the 50 most common mutations in the *TP53* gene are associated with disruption of protein structures and highly deleterious VIPUR scores (> 0.5)[40]. They demonstrated that *R175H* (exon 5) mutation had the highest frequency, whereas *R248Q* (exon 7) and *R273H* (exon 8) mutations were located in the next positions.

No studies were found on the status of the *KRAS* and *DDR2* genes in the Iranian population [41]. To date, we have only found two studies on *TP53* mutations in SCC, however, other subtypes (ADC, LCC, SCLC) have not yet been evaluated in Iranian patients [42, 43]. The above-mentioned lines of evidence and geographical variation in the prevalence of gene mutations indicate that studying the status of *KRAS*, *DDR2*, and *TP53* may have important implications for diagnosis, prognosis, cancer recurrence prevention, and designing clinical trials and targeted therapies for Iranian population with lung cancer. Therefore, we conducted this study to explore the status of *KRAS*, *DDR2*, and *TP53* genes in hotspot regions on a panel of lung cancer samples including three major NSCLC subtypes (ADC, SCC, and LCC) and SCLC in the Iranian population. Moreover, we examined the potential correlations among mutational status of *KRAS*, *DDR*, and *TP53* genes with clinicopathological parameters in this study.

## Materials and methods

### Patient characteristics

Fifty-five formalin-fixed paraffin-embedded (FFPE) samples of lung cancer, including NSCLC and SCLC, were collected from several referral hospitals in Tehran, Iran. All samples were investigated by an expert pathologist and had a histologic diagnosis of primary lung carcinoma, containing at least 50% tumor cells [44, 45]. We selected cases with sufficient material for molecular analyses. The specimens were obtained before any systematic treatment. The clinicopathological parameters of the patients, including tumor types, histological grade (in SCC and ADC) and inflammation (in SCC) were obtained by reviewing their medical records. This research was approved by the Iran University of Medical Sciences (IUMS) Research Ethics Committee. Patients’ data were kept fully anonymous.

### Mutational analysis

#### DNA extraction

After removing the surrounding paraffin, the tissues were cut into seven micrometer thick sections, xylene (Merck Co., Germany) was added and the samples were incubated at 60°C for deparaffinization. The samples were then hydrated with a decreased serial dilution of ethanol and incubated at 60°C at each step. A lysis solution and proteinase K were added to the tissue samples, which were then incubated at 60°C overnight. DNA extraction was performed using the FavorPrep™ Tissue Genomic DNA Extraction Mini Kit (Cat number: FATGK001, Favorgen, Taiwan) following the manufacturer’s recommendations. Extracted DNA was quantified on the NanoDrop 8000 (Thermo Scientific).

#### PCR

PCR was carried out using a super PCR Master Mix 2X (Cat number: YT1553, Yekta Tajhiz Azma Co., Iran) according to the manufacturer’s protocol. The PCR program, which was repeated for each gene over 35 cycles, was as follows; 94°C for one minute, annealing phase at 57.5°C for *KRAS*, 55.5°C for *DDR2* and 52°C for *TP53* for one minute, and extension phase at 72°C for three minutes. The PCR products were electrophoresed on a 1% agarose gel.

Most of the tumor samples collected were of the SCC type and *S768R* substitutions are commonly reported in SCC patients. As such, we decided to design the primers for exon 18 of *DDR2* to be 5’-GGGTATAGCTGCAGATTATGAA-3´ for forward and 5´-CATTCATCCCCAACAGTTCTTA-3´ for reverse. Primers were designed by an online website (http://simgene.com/Primer3). We also used the previously described primer pairs (5’-TTTCTTTGCTGCCGTCTTC-3´ as forward and 5´-TTGCACATCTCATGGGGTTA-3´ as reverse) for exons 5–6 of *TP53* and 5’-AAAGGTACTGGTGGAGTATTTGATAGTG-3´ as forward and 5´-TCATGAAAATGGTCAGAGAAACCT-3´ as reverse primers for exon 2 (codon 12) of *KRAS* (23, 24). To confirm the quality of these primers, we examined the number of nucleotides, Tm temperature, GC ratio, the possibility of forming secondary structures and their proper attachment to the desired gene with the help of online tools, such as the NCBI Primer BLAST (www.ncbi.nlm.nih.gov/tools/primer-blast/) and the Beacon Designer program (http://www.premierbiosoft.com/). All the primers are listed in S1 Table. We also included appropriate negative control at each PCR process, as mentioned in the Sanger sequencing guidelines [46].

#### Sequencing

After confirming the band for each gene, the PCR products were purified and screened for mutations using the Sanger sequencing analysis (DNA Analyzer ABI PRISM® 3700).

### Data analysis

Statistical analyses were performed using SPSS software version 20 (SPSS, Chicago, IL, USA). The associations of *TP53* status with clinicopathological parameters were assessed using Pearson’s χ 2 or the Fisher's exact test, where appropriate. A p- value of < 0.05 was considered statistically significant.

All sequences were analyzed by mutation surveyor V3.30 (Softgenetics, Pennsylvania, US). Quality scores for all the sequences were more than 20, with less than 5% noise. We only confirmed the variations that did not have noise in the region of interest (ROI) of the sequence. We excluded the sequences without these criteria. We also evaluated the quality of the sequences and variations by Sequence Scanner v1.0. For each variant, Phred scores were more than 30 (between 48 to 62). The variants were described in HGVS nomenclature (GRCh38) by an online tool (https://mutalyzer.nl). We also used the following in-silico tools, as described in the ACMG guidelines [47, 48], for interpretation of sequence variants: MutationTaster (http://www.mutationtaster.org/), CADD (http://cadd.gs.washington.edu/), varsome (https://varsome.com/) and CGI (https://www.cancergenomeinterpreter.org). For checking the previously reported variants, we reviewed the following online databases: The UniProt database (http://www.uniprot.org; UniProtKB ID Q8IYM9), the NCBI dbSNP database (https://www.ncbi.nlm.nih.gov/SNP/), the Catalogue of Somatic Mutations in Cancer (COSMIC; http://cancer.sanger.ac.uk/cosmic) and 1000 Genomes (http://www.1000genomes.org/).

## Results and discussion

### Study population

Fifty-five tumor samples were used for the *KRAS* gene mutation analysis. Due to a lack of genomic DNA, *TP53* and *DDR2* were examined in 44 and 46 samples, respectively. The patient characteristics are summarized in S2 Table. The median age of the patients was 65.7 years (range, 37–83 years). They were 46 male (83.6%) and 9 female patients (16.36%) (male to female ratio = 5.1). Thirteen patients (23.63%) had ADC, 34 (61.81%) SCC, four (7.27%) LCC, one (1.81%) mixed LCC/SCC, two (3.63%) NSCLC without mentioned subtype and one (1.81%) SCLC. SCC was the major histologic type. The histologic grade of patients was as follows: three (23.07%): poor, four (30.76%): moderate, and two (15.38%): well differentiated in ADC, and 10 (26.41%): poor, six (17.64%): moderate, and 13 (38.23%): well differentiated in SCC. A total of 12 (35.29%) patients with SCC had inflammation.

### *KRAS* Mutations analysis

**Fig 1:**
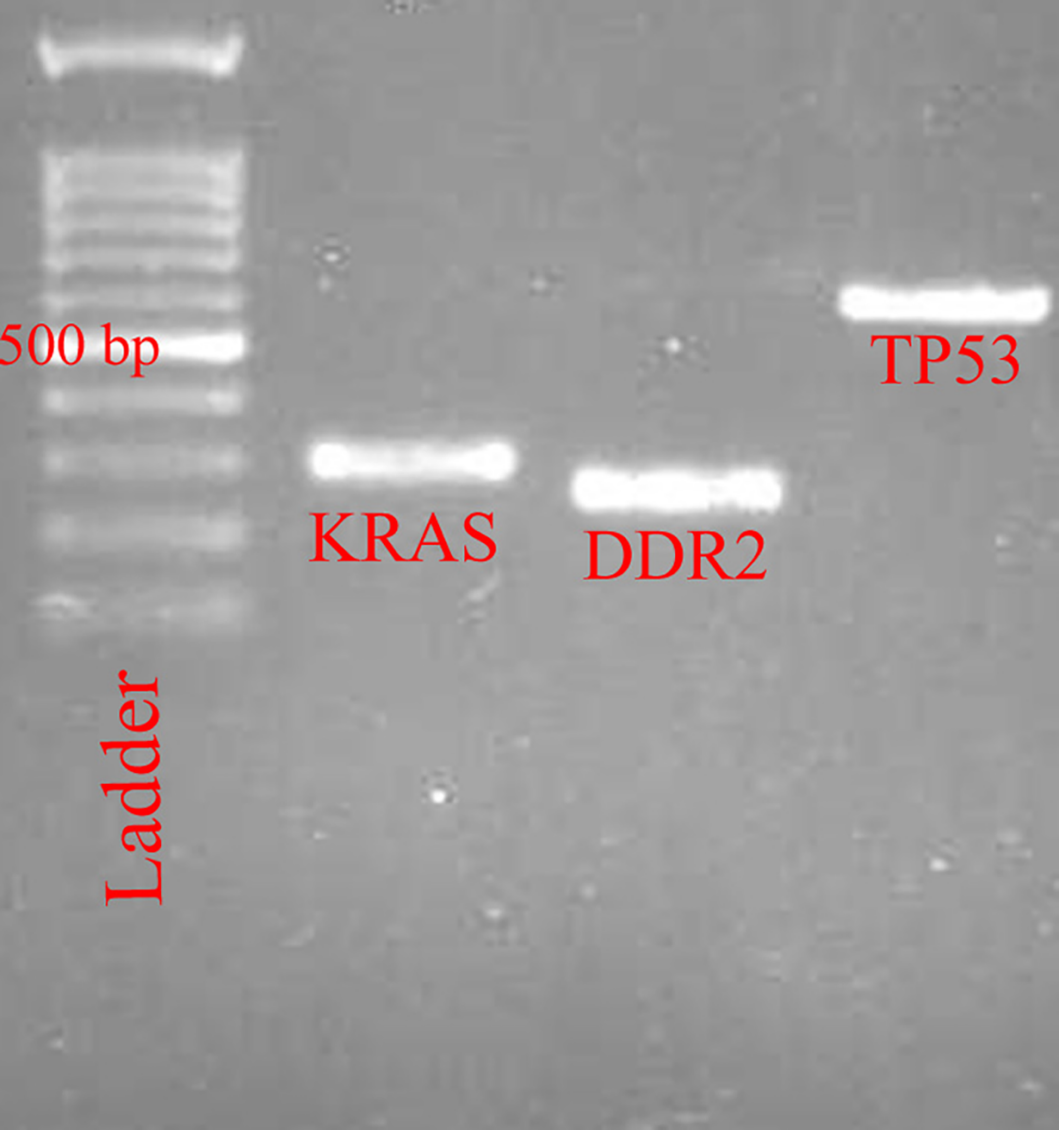
The PCR products on the gel agarose electrophoresis.

**Fig 2:**
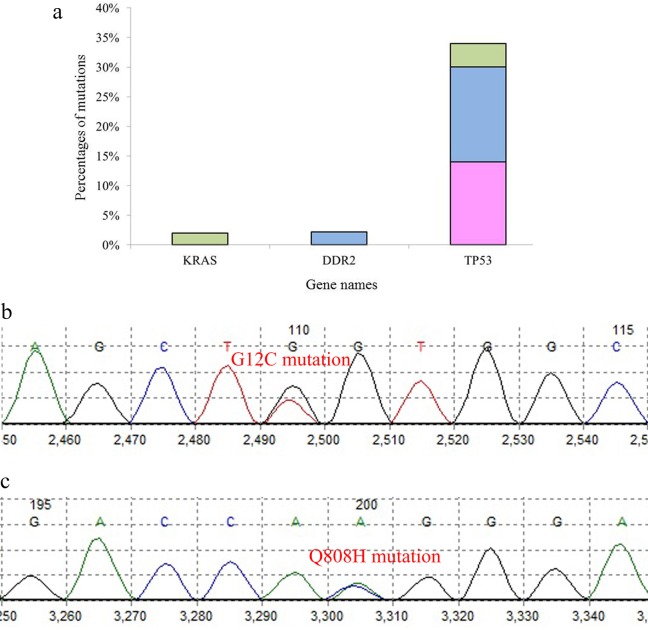
Analysis of the lung cancer samples for *KRAS*, *DDR2* and *TP53* gene mutations. (a) percentage of *KRAS*, *DDR2* and *TP53* mutations in different subtypes of lung cancer; (green: SCLC, blue: SCC, pink: ADC). (b) *G12C* mutation in *KRAS* and (c) *Q808H* mutation in *DDR2*.

*G12C* substitution, with G>T transversion (GGT>TGT), was observed in a 67-year-old man with LCC (2%), but there was no other mutation in patients with ADC and SCC (Figs 1 and 2). An rs1625895 variant was another finding in the *TP53* gene of this patient (Tables 1–4).

**Table 1:** Frequency and type of coding variants in *KRAS*, *DDR2*, and *TP53* genes in lung tumor samples.

Pt. ID	Gender	Age	Tumor type	*KRAS*	*DDR2*	*TP53*	Phred Score (ROI)
8	M	67	SCC	No.	No.	*D186G*	52
						*H193R*	51
						*P222P*	50
13	M	69	SCC	No.	No.	*Q167Q*	48
15	M	67	SCC	No.	*Q808H*	NA	51
25	M	58	ADC	No.	No.	*V157F*	54
26	M	67	ADC	No.	No.	*D186G*	55
						*H193R*	52
						*P222P*	51
35	M	45	SCC	No.	No.	*F212L*	62
36	M	64	SCC	No.	No.	*F212L*	50
41	M	72	SCC	No.	No.	*V147A*	50
49	F	68	ADC	NA	No.	*T211T*	62
						*P222P*	62
67	M	62	LCC	No.	No.	*D186G*	53
						*H193R*	53
70	M	67	LCC	*G12C*	No.	No.	57

M = male, F = female, ROI = region of interest

NA = Not available

**Table 2:** The data of observed variations based on HGVS38 in coding sequence.

Transcript ID	RefSeq	Gene	Gene role	Variant	HGVS38 (Chromosomal variant)	HGVS38 (transcripts variant)	MAF	db SNP ID/ COSMIC ID
ENST00000311936.7	NM_004985	KRAS	OG	G12C	NC_000012.12:g.25245351C>T	NM_004985.4:c.34G>A	1.976e-05	rs121913530
ENST00000367922.7	NM_001014796	DDR2	OG	Q808H	NC_000001.11:g.162778720A>C	NM_001014796.1:c.2424A>C	0.0002393	rs765660823
ENST00000269305.8	NM_000546	TP53	TSG	D186G	NC_000017.11:g.7675055T>C	NM_000546.5:c.557A>G	NM.	COSM46287
				H193R	NC_000017.11:g.7674953T>C	NM_000546.5:c.578A>G	NM.	rs786201838
				P222P	NC_000017.11:g.7674865C>T	NM_000546.5:c.666G>A	6.748e-05	rs72661118
				Q167Q	NC_000017.11:g.7675111C>T	NM_000546.5:c.501G>A	NM.	COSM44299
				V157F	NC_000017.11:g.7675143C>A	NM_000546.5:c.469G>T	0.00006/7	rs121912654
				F212L	NC_000017.11:g.7674897A>G	NM_000546.5:c.634T>C	NM.	COSM45477
				V147A	NC_000017.11:g.7675172A>G	NM_000546.5:c.440T>C	NM.	COSM45819
				T211T	NC_000017.11:g.7674898A>G	NM_000546.5:c.633T>C	NM.	COSM46211

OG: Oncogene, TSG: Tumor Suppressor Gene, MAF: Minor Allele Frequency, NM: Not Mention.

**Table 3:** The data of observed variations based on HGVS38 in non-coding sequence.

gene	HGVS38 (Chromosomal variant)	HGVS38 (transcripts variant)	db SNP ID/ COSMIC ID	Mutation taster
TP53	NC_000017.11:g.7674983T>C	NM_000546.5:c.560-12A>G	Novel	Polymorphism
	NC_000017.11:g.7674773A>G	NM_000546.5:c.672+86T>C	Novel	Polymorphism
	NC_000017.11:g.7674853C>T	NM_000546.5:c.672+6G>A	rs766856111	Polymorphism
	NC_000017.11:g.7674797T>C	NM_000546.5:c.672+62A>G	rs1625895	Polymorphism

**Table 4:** The predictions of variants effect based on in silico tools.

Gene	Variant	Cadd phred	Raw Score	Oncogenic classification*	Mutation taster	SIFTcat	PolyPhenCat	DANN score	ClinVar
*KRAS*	*G12C*	31	6.5	NSCLC; OV; LUAD; THCA; COREAD;	Disease causing	Deleterious	Possibly damaging	0.9987	Pathogenic
*DDR2*	*Q808H*	24.7	4.7	TIER 2	Disease causing	Deleterious	Probably damaging	0.9953	NM
*TP53*	*D186G*	22.9	3.3	Passenger	Disease causing	Tolerated	Probably damaging	0.9943	NM
	*H193R*	23.5	3.9	known in any cancer type	Disease causing	Deleterious	Probably damaging	0.9876	Likely pathogenic
	*P222P*	21.2	2.7	Not protein affecting	Disease causing	NM	NM	0.5001	Likely benign
	*Q167Q*	8.331	0.6	Not protein affecting	Disease causing	NM	NM	0.5293	NM
	*V157F*	24.2	4.4	Hepatocellular carcinoma	Disease causing	Deleterious	Probably damaging	0.9909	Pathogenic/Likely pathogenic
	*F212L*	10.92	1.0	TIER 1	Polymorphism	Tolerated	Benign	0.7743	NM
	*V147A*	25.9	5.3	TIER 1	Disease causing	Deleterious	Probably damaging	0.9917	NM
	*T211T*	3.703	0.1	Not protein affecting	Disease causing	NM	NM	0.487	NM

* https://www.cancergenomeinterpreter.org

According to the oncodriveMUT method (tier 1 and 2 represent higher and lower level of stringency of the driver prediction, respectively).

NSCLC: Non-small Cell Lung Cancer, OV: Ovary Cancer, LUAD: Lung Adenocarcinoma, THCA: Thyroid Carcinoma, COREAD: Colorectal Adenocarcinoma, NM: Not Mention.

### *DDR2* mutations analysis

*Q808H* substitution with A>C transversion (CAA>CAC) was observed in the SCC tumor specimen of a 67-year-old male (2.17%; Figs 1 and 2), but no mutation was seen in the ADC and LCC subtypes (Tables 1–4).

### *TP53* mutations analysis

**Fig 3:**
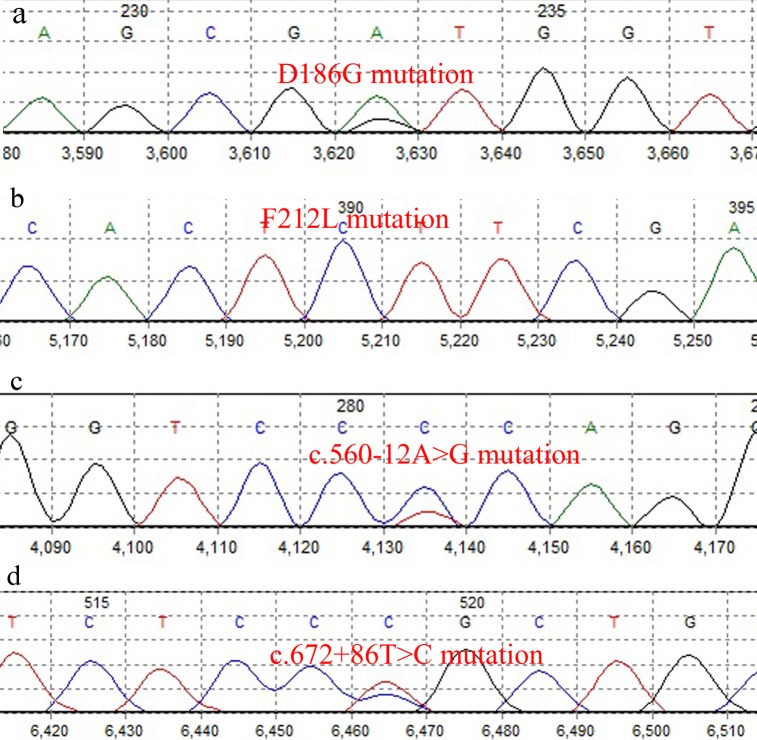
Analysis of *TP53* gene mutations in lung cancer samples. (a) *D186G* mutation in coding sequence (b) *F212L* mutation in coding sequence (c) *c*.*560-12A>G* mutation in intronic region (d) *c*.*672+86T>C* mutation in intronic region.

We found 48 variants in 35 of the 44 (79.54%) patients, in which 15 variants (31.25%) were in coding regions and 33 variants (68.75%) were intronic. *V147A*, *V157F*, *Q167Q*, *D186G*, *H193R*, *T211T*, *F212L* and *P222P* were the coding variants which were detected in nine patients (20.45%), including six (13.63%) in ADC, seven (15.9%) in SCC and two (4.54%) in LCC (Figs 1 and 3). The most frequently mutated sites were codons 186 (n = 3) and 193 (n = 3) with A>G transition, codon 222 (n = 3) with G>A transition and codon 212 (n = 2) with T>C transition. G>A transition in codon 167, G>T transversion in codon 157, T>C transition in codon 147 and T>C transition in codon 211 were other mutated base sites in *TP53*. Among all patients, the A>G transition was the most frequent (n = 6, 40%) base change in the coding region. Previously identified as polymorphism, rs1625895 (HGVS: *c*.*672+62A>G*) was a frequent intronic variant in 31 of the 44 patients (70.45%). rs766856111 (HGVS: *c*.*672+6G>A*) was another intronic variant in the SCC tumor sample of a 61-year-old male with intensive inflammation. We also found two new variants in two SCC male patients. *c*.*560-12A>G* was found in a 72-year-old patient and *c*.*672+86T>C* in a 37-year-old patient (Tables 1–4).

The exploration of *TP53* status and clinicopathologic factors revealed a relative positive correlation between the presence of mutation in *TP53* with age (*P* = 0.08)(Table 5). The correlations between *TP53* status and other clinicopathological parameters are summarized in Table 5.

**Table 5:** Correlations between *TP53* mutational status and clinicopathological parameters.

Characteristics			Total number (%)	*TP53* mutant	*TP53* wild-type	P-value
Age, year	≤ 65 years		18(41)	12(67)	6(33)	0.08
	> 65 years		26(59)	23(89)	3(11)	
Gender	Male		37(84)	29(78)	8(22)	0.55
	Female		7(16)	6(86)	1(14)	
Tumor type	NSCLC	ADC	13(30)	10(77)	3(23)	0.13
		SCC	24(55)	18(75)	6(25)	
		LCC	3(7)	3(100)	0(0)	
		LCC/SCC	1(2)	1(100)	0(0)	
		NM	2(4)	2(100)	0(0)	
	SCLC	SCLC	1(2)	1(100)	0(0)	
Histological Grade	ADC	Well	2(22)	1(50)	1(50)	0.56
		Moderate	4(45)	3(75)	1(25)	
		Poor	3(33)	2(67)	1(33)	
	SCC	Well	9(43)	6(67)	3(33)	0.49
		Moderate	5(24)	5(100)	0(0)	
		Poor	7(33)	5(71)	2(29)	
Inflammation (SCC)	Yes		0(0)	6(67)	3(33)	0.54
	No		0(0)	4(80)	1(20)	

NSCLC = non-small cell lung cancer, SCLC = small cell lung cancer, ADC = adenocarcinoma, SCC = squamous cell carcinoma, LCC = large cell carcinoma.

NM = not mention.

As previously established, lung cancer is the second most common and the most lethal type of cancer [49]. In Iran, lung cancer is the second cause of cancer-related death, after stomach malignancies [50]. Surgery, radiotherapy and chemotherapy are some of the commonly used treatments for lung cancer and are often used in the early stages of lung cancer. For some cases of lung cancer, targeted therapies or immunotherapy can also be used. Due to the toxicity of some medications, and the lack of response in some patients to common treatments, researchers face serious challenges in the treatment of lung cancer [51, 52]. A review of the genetic variations of lung cancer can be effective in detecting the disease as quickly as possible and choosing an effective treatment.

For the first time, this study was designed to investigate the status of *KRAS*, *DDR2*, and *TP53* in hotspot regions in a panel of lung cancer, including NSCLC and SCLC, in Iranian population. In addition, we examined the association between mutational status of these genes and clinicopathological parameters.

Almost 15% to 25% of patients with NSCLC have *KRAS* mutations [27]. These mutations occur more frequently in ADC (approximately 30%) and less frequently in the SCC subtype (approximately 5%). More than 97% of *KRAS*-mutant cases affect exon 2 (*G12*, *G13*), which disrupts common targeted therapies in lung cancer [53, 54]. Therefore, targeted therapies have been provided based on the *KRAS* mutations (S3 Table). In this study, 23.63% of patients were diagnosed with ADC and 61.81% with SCC, but the *G12C* variant was seen in LCC, which contained only 7.27% of the tumor samples. There is evidence that the frequency of *KRAS* mutations in ADC varies among different ethnic groups, with a lower frequency observed among Asians compared to Caucasians [55]. Mutation frequency of *KRAS* in Chinese, Japanese, and Korean populations with ADC was 5.7% (range: 0.0%– 18.2%), 11.3% (range: 6.6%– 14.2%), and 9% (range: 7.3%– 9.5%), respectively, whereas this amount was 28.1% in Europe [55]. It can be concluded that the frequency of *KRAS* mutations in Iran, as a Western Asian country, may vary from 0 to 1.8 per 10 patients with ADC (0/10 to 1.8/10). These findings may indicate a different distribution of *KRAS* mutations in patients with lung cancer in the Iranian population.

We also examined the status of *DDR2*, which has been reported as a variable factor in lung cancer, most commonly in SCC, with a frequency of 3.8%. The mutations in this gene do not correlate with the gender or age of patients [10]. *DDR2* mutations have been observed in conjunction with the *KRAS* (*G12C*) mutation [56].

Our results showed *Q808H* substitution (rs765660823) with A>C transversion (CAA>CAC) in SCC samples, which was previously reported by Exome Aggregation Consortium. This variant is in the tyrosine kinase domain (563–849) of *DDR2*, which may result in hyper-activation of this oncogene [57]. We investigated the COSMIC to find this variant in different cancers. However, there were no journal citations for this particular variant. Thus, we searched the Greater Middle East (GME) Variome Project (http://igm.ucsd.edu/gme/) website but still did not find any reported data on this variant in the countries of the Greater Middle East. Analysis using in silico tools, such as MutationTaster and CADD, revealed disease-causing and pathogenic effects for this variant, which is categorized as tier II, with potential clinical significance in CGI. No study was conducted about this variant, and to the best of our knowledge, our study was the first to report this variant in a cancer study.

As a tumor suppressor gene, *TP53* is reported as the most mutated gene in lung cancer and its mutations are observed in 50% of NSCLC and 65% of SCC cases, which is higher than in ADC [58]. In the current study, all variations, including benign, pathogenic or intermediate, in coding and intronic sequences of hotspot regions of *TP53* have been reported, using Oncogenic classification (https://www.cancergenomeinterpreter.org) that is a reliable database [59, 60] (Table 4). In our study, *TP53* variants were observed in 79.54% of the samples, including 31.25% in conding and 68.75% in intronic regions, and had the highest frequency of variations among the three genes. Coding variants *V147A*, *V157F*, *Q167Q*, *D186G*, *H193R*, *T211T*, *F212L* and *P222P* were detected in nine patients (20.45%). Among these variants, *V147A*, *V157F* and *H193R* were already documented in lung cancers [61–63]. *D186G* and *F212L* were reported in some malignant tumors, including ADC of large intestine and maxillary sinus SCC, but we did not find any reported data about these variants in lung cancer [64, 65](S4 Table).

Chromatogram study of patient 35 showed homozygous mutations in *F212L*. Considering that the age of the patient was less than the mean age of patients with lung cancer, it may be possible that lung cancer in this patient was familial. Unfortunately, the patient died at the time of the study and samples of blood or other tissues were not available. The patient's family was not able to be located for supplemental studies. rs1625895 (HGVS: *c*.*672+62A>G*) was a frequent intronic polymorphism in our study, seen in 31 of the 44 patients (70.45%). A significant association between TP53 intron 6 variant (rs1625895) with increased risk of lung cancer has been reported [66].

The association of *TP53* status and clinicopathological parameters revealed a marginal trend between the presence of *TP53* mutation and older age. However, no data exist in the literature on the association of *TP53* status and clinicopathological characteristics in the Iranian population with lung cancer [42, 43].

Many researchers have claimed that mutations in *TP53* are prognostic, or predictive, to treatment response, while others have failed to demonstrate this association [36, 67]. Since most chemo-therapeutics induce DNA damage and consequently activate the p53 protein, mutations in the *TP53* gene can negatively affect responses to this treatment [68]. In addition, cancer stem cells (CSCs) within the tumors are one of the reasons for resistance to treatment, relapse, and metastasis of the tumors. It is suggested that the level of expression of these genes be evaluated with important indicators of the CSC population including CD44, CD133, and ALDH1 in subsequent studies [69, 70]. Moreover, conducting large population-based studies is highly recommended.

Application of the next generation sequencing (NGS) will help increase sensitivity and quality of data in finding mutation(s), but selecting a method is determined by several factors including sample type (fresh, frozen, or FFPE), quality and quantity of DNA, or RNA [71]. The PCR-based enrichment is the most preferred methodology for FFPE samples, which can efficiently amplify targeted regions of interest for sequencing analysis from low amounts of FFPE DNA; thus, the direct DNA sequencing methods, such as Sanger sequencing, are still accepted as the gold standard for mutations diagnosis [72]. In addition, to improve the sensitivity of molecular analysis, a pathologist can be asked to evaluate the tissue samples using a microscope to select a suitable area with high tumor cells proportion. Thus, in the present survey, we selected lung tumor samples containing at least 50% tumor cells [44, 45].

## Conclusions

In conclusion, *KRAS*, *DDR2*, and *TP53* variants were detected in 2%, 2.17% and 79.54% of all cases, respectively. The frequency of *DDR2* mutation is nearly close to other studies, while *KRAS* and *TP53* mutation frequencies are lower and higher than other populations, respectively. Three new putative pathogenic variants, for the first time, have been detected in Iranian patients with lung cancer, including *Q808H* in *DDR2*, *F212L*, and *D186G* in coding regions of *TP53*. In addition, we observed five novel benign variants, including *Q167Q*, *P222P* and *T211T* in coding sequence, and *c*.*560-12A>G* and *c*.*672+86T>C*, in intronic region of *TP53*. Mutations of *KRAS* and *DDR2* were found in LCC and SCC subtypes, respectively, whereas mutations of *TP53* were seen in SCC and ADC subtypes with higher frequencies and LCC subtype with lower frequency. Therefore, Iranian lung cancer patients can benefit from mutational analysis before starting the conventional treatment. A better understanding of the biology of these genes and their mutations will be critical for developing future targeted therapies.

## Supporting information

S1 Table The sequence of primers *KRAS, DDR2* and *TP53* genes. (DOC)

S2 Table Clinicopathological characteristics of lung cancer patients. (DOC)

S3 Table Drugs for *G12C* mutation of gene *KRAS* in lung cancer patients in cancer genome interpreter*. * https://www.cancergenomeinterpreter.org.
(DOC)

S4 Table Alterations described as biomarkers for *TP53* in different tumor types in cancer genome interpreter*. LIP: Liposarcoma, HNC: Head and neck cancer, BRCA: Breast Adenocarcinoma, BCL: B cell Lymphoma, FGCT: Female Germ Cell Tumor, MGCT: Male Germ Cell Tumor, AML: Acute Myeloid Leukemia, MDPS: Myelodysplastic Proliferative Syndrome, OV: Ovary Cancer, THYM: Thymic.
* https://www.cancergenomeinterpreter.org
(DOC)
