# Supplementary material for: Distribution of KRAS, DDR2, and TP53 gene mutations in lung cancer: An analysis of Iranian patients
Source: PLoS One. 2018 Jul 26;13(7):e0200633. doi: 10.1371/journal.pone.0200633 (PMC6061986; doi:10.1371/journal.pone.0200633)
Supplement: S1 Table — (DOC) [file pone.0200633.s001.doc]

**S1 Table. The sequence of primers *KRAS*, *DDR2* and *TP53* genes.**

| **Name of genes** | **Accession Number** | **Primers (5ꞌ→3ꞌ)** | **GC %** | **GC Clamp** | **Cross Dimer (ΔG)** | **Self Dimer (ΔG)** | **Hairpin (ΔG)** | **Annealing Tm (°C)** | **PCR Product length (bp)** |
| --- | --- | --- | --- | --- | --- | --- | --- | --- | --- |
| *KRAS* | NG_007524.1 | Forward: AAAGGTACTGGTGGAGTATTTGATAGTG | 39.29 | 1 | -2.7 | -2.0 | -1.5 | 57.5 | 291 |
|  |  | Reverse: TCATGAAAATGGTCAGAGAAACCT | 37.5 | 1 | -2.7 | -4.9 | -1.5 |  |  |
| *DDR2* | NG_016290.2 | Forward: GGGTATAGCTGCAGATTATGAA | 40.91 | 1 | -3.0 | -6.5 | 0.5 | 55.5 | 258 |
|  |  | Reverse: CATTCATCCCCAACAGTTCTTA | 40.91 | 1 | -3.0 | -0.7 | 0.0 |  |  |
| *TP53* | NG_017013.2 | Forward: TTTCTTTGCTGCCGTCTTC | 47.37 | 1 | -2.0 | 0.0 | 0.0 | 52 | 588 |
|  |  | Reverse: TTGCACATCTCATGGGGTTA | 45 | 1 | -2.0 | -3.4 | -0.5 |  |  |
