# Supplementary material for: Distribution of KRAS, DDR2, and TP53 gene mutations in lung cancer: An analysis of Iranian patients
Source: PLoS One. 2018 Jul 26;13(7):e0200633. doi: 10.1371/journal.pone.0200633 (PMC6061986; doi:10.1371/journal.pone.0200633)
Supplement: S2 Table — (DOC) [file pone.0200633.s002.doc]

S2 Table. Clinicopathological characteristics of lung cancer patients.

| **Characteristics** | | | **Number (%)** |
| --- | --- | --- | --- |
| **Age, year** | ≥ 65 years | | 36 (65.45%) |
|  | < 65 years | | 19 (34.54%) |
| **Gender** | Male | | 46 (83.6%) |
|  | Female | | 9 (16.36%) |
| **Tumor type** | NSCLC | AD | 13 (23.63%) |
|  |  | SCC | 34 (61.81%) |
|  |  | LCC | 4 (7.27%) |
|  |  | LCC/SCC | 1 (1.81%) |
|  |  | NM. | 2 (3.63%) |
|  | SCLC | SCLC | 1 (1.81%) |
| **Histological Grade** | ADC | Well | 2 (15.38%) |
|  |  | Moderate | 4 (30.76%) |
|  |  | Poor | 3 (23.07%) |
|  |  | NM. | 4 (30.76%) |
|  | SCC | Well | 13 (38.23%) |
|  |  | Moderate | 6 (17.64%) |
|  |  | Poor | 10 (26.41%) |
|  |  | NM. | 4 (11.76%) |
| **Inflammation (SCC)** | Yes | | 12 (35.29%) |
|  | No | | 7 (20.58%) |
|  | NM. | | 15 (44.11%) |

NSCLC=non-small cell lung cancer, SCLC=small cell lung cancer, ADC= adenocarcinoma, SCC=squamous cell carcinoma, LCC= large cell carcinoma.

NM= not mention.
