# Supplementary material for: Distribution of KRAS, DDR2, and TP53 gene mutations in lung cancer: An analysis of Iranian patients
Source: PLoS One. 2018 Jul 26;13(7):e0200633. doi: 10.1371/journal.pone.0200633 (PMC6061986; doi:10.1371/journal.pone.0200633)
Supplement: S3 Table — * https://www.cancergenomeinterpreter.org. (DOC) [file pone.0200633.s003.doc]

**S3 Table. Drugs for *G12C* mutation of gene *KRAS*** in lung cancer patients in cancer genome interpreter*.

| **Drug** | **Effect** | **Evidence** |
| --- | --- | --- |
| Selumetinib (MEK inhibitor) | No Responsive | Early trials |
| Panitumumab (EGFR mAb inhibitor) | Resistant | NCCN guidelines |
| Cetuximab (EGFR mAb inhibitor) | Resistant | NCCN guidelines |
| BET inhibitors | Responsive | Pre-clinical |
| CDK4 inhibitors | Responsive | Pre-clinical |
| CDK4/6 inhibitor + MEK inhibitors | Responsive | Early trials |
| FAK inhibitors | Responsive | Pre-clinical |
| FAS inhibitors | Responsive | Case report |
| FAS inhibitors | Responsive | Case report |
| HSP90 inhibitor (in combination)s | Responsive | Pre-clinical |
| JAK/TBK1/IKKµ inhibitors | Responsive | Pre-clinical |
| MEK inhibitors | Responsive | Early trials |
| pan-RAF inhibitors | Responsive | Early trials |
| PI3K pathway inhibitor + MEK inhibitors | Responsive | Early trials |
| Abemaciclib (CDK4/6 inhibitor) | Responsive | Early trials |

* https://www.cancergenomeinterpreter.org
