# Supplementary material for: Distribution of KRAS, DDR2, and TP53 gene mutations in lung cancer: An analysis of Iranian patients
Source: PLoS One. 2018 Jul 26;13(7):e0200633. doi: 10.1371/journal.pone.0200633 (PMC6061986; doi:10.1371/journal.pone.0200633)
Supplement: S4 Table — LIP: Liposarcoma, HNC: Head and neck cancer, BRCA: Breast Adenocarcinoma, BCL: B cell Lymphoma, FGCT: Female Germ Cell Tumor, MGCT: Male Germ Cell Tumor, AML: Acute Myeloid Leukemia, MDPS: Myelodysplastic Proliferative Syndrome, OV: Ovary Cancer, THYM: Thymic. * https://www.cancergenomeinterpreter.org (DOC) [file pone.0200633.s004.doc]

**S4 Table. Alterations described as biomarkers for *TP53*** in different tumor types in cancer genome interpreter*.

| **Drug** | **Effect** | **Tested Tumor** | **Evidence** | **TP53 mutations** |
| --- | --- | --- | --- | --- |
| MDM2 inhibitors | Resistant | LIP | Early trials | D186G, H193R, V157F, F212L, V147A, |
| WEE1 inhibitors | Responsive | HNC | Pre-clinical | D186G, H193R, F212L, V147A, |
| Abemaciclib (CDK4/CDK6 inhibitor) | Resistant | BRCA | Early trials | D186G, H193R, V157F, F212L, V147A, |
| AZD6738 (ATR inhibitor) | Responsive | BCL | Early trials | D186G, H193R, F212L, V147A, |
| Cisplatin (Chemotherapy) | Resistant | FGCT, MGCT | Early trials | D186G, H193R, V157F, F212L, V147A, |
| Decitabine (Chemotherapy) | Responsive | AML, MDPS | Early trials | D186G, H193R, V157F, F212L, V147A, |
| Doxorubicin (Anthracycline antitumor antibiotic) | Responsive | BLCA | Pre-clinical | D186G, H193R, V157F, F212L, V147A, |
| Gemcitabine (Chemotherapy) | Responsive | BLCA | Pre-clinical | D186G, H193R, V157F, F212L, V147A, |
| Mitomycin C (Chemotherapy) | Responsive | BLCA | Pre-clinical | D186G, H193R, V157F, F212L, V147A, |
| MK-1775 (WEE1 inhibitor) | Responsive | OV | Early trials | D186G, H193R, V157F, F212L, V147A, |
| Pramlintide (Amylin analogue) | Responsive | THYM | Pre-clinical | D186G, H193R, V157F, F212L, V147A, |

LIP: Liposarcoma, HNC: Head and neck cancer, BRCA: Breast Adenocarcinoma, BCL: B cell Lymphoma, FGCT: Female Germ Cell Tumor, MGCT: Male Germ Cell Tumor, AML: Acute Myeloid Leukemia, MDPS: Myelodysplastic Proliferative Syndrome, OV: Ovary Cancer, THYM: Thymic.

* https://www.cancergenomeinterpreter.org
